# Delineation of Spatial Variability in the Temperature–Mortality Relationship on Extremely Hot Days in Greater Vancouver, Canada

**DOI:** 10.1289/EHP224

**Published:** 2016-06-27

**Authors:** Hung Chak Ho, Anders Knudby, Blake Byron Walker, Sarah B. Henderson

**Affiliations:** 1 Department of Geography, Simon Fraser University, Burnaby, British Columbia, Canada; 2 Institute of Environment, Energy and Sustainability, Chinese University of Hong Kong, Hong Kong; 3 Department of Geography, University of Ottawa, Ontario, Canada; 4 British Columbia Centre for Disease Control, Vancouver, British Columbia, Canada; 5 School of Population and Public Health, University of British Columbia, Vancouver, British Columbia, Canada

## Abstract

**Background:**

Climate change has increased the frequency and intensity of extremely hot weather. The health risks associated with extemely hot weather are not uniform across affected areas owing to variability in heat exposure and social vulnerability, but these differences are challenging to map with precision.

**Objectives:**

We developed a spatially and temporally stratified case-crossover approach for delineation of areas with higher and lower risks of mortality on extremely hot days and applied this approach in greater Vancouver, Canada.

**Methods:**

Records of all deaths with an extremely hot day as a case day or a control day were extracted from an administrative vital statistics database spanning the years of 1998–2014. Three heat exposure and 11 social vulnerability variables were assigned at the residential location of each decedent. Conditional logistic regression was used to estimate the odds ratio for a 1°C increase in daily mean temperature at a fixed site with an interaction term for decedents living above and below different values of the spatial variables.

**Results:**

The heat exposure and social vulnerability variables with the strongest spatially stratified results were the apparent temperature and the labor nonparticipation rate, respectively. Areas at higher risk had values ≥ 34.4°C for the maximum apparent temperature and ≥ 60% of the population neither employed nor looking for work. These variables were combined in a composite index to quantify their interaction and to enhance visualization of high-risk areas.

**Conclusions:**

Our methods provide a data-driven framework for spatial delineation of the temperature-–mortality relationship by heat exposure and social vulnerability. The results can be used to map and target the most vulnerable areas for public health intervention.

**Citation:**

Ho HC, Knudby A, Walker BB, Henderson SB. 2017. Delineation of spatial variability in the temperature–mortality relationship on extremely hot days in greater Vancouver, Canada. Environ Health Perspect 125:66–75; http://dx.doi.org/10.1289/EHP224

## Introduction

Climate change has increased the severity, intensity, and frequency of extreme hot weather events over the past century (Meehl and Tebaldi 2004; Reid et al. 2009). Many of these events have been associated with excess mortality in urban areas, as documented in the United States (Curriero et al. 2002; Kaiser et al. 2001; Semenza et al. 1996), Europe (Filleul et al. 2006; Schifano et al. 2009), China (Huang et al. 2010), and Russia (Trenberth and Fasullo 2012). Less severe impacts have also been documented in the Canadian cities of Montreal (Smargiassi et al. 2009), Toronto (Pengelly et al. 2007), and Vancouver (Kosatsky et al. 2012), the last being the setting for our study. Cities are at particular risk during extreme hot weather events owing to the urban heat island (UHI) effect, which leads to intra-urban variability in heat exposure (Hart and Sailor 2009; Hawkins et al. 2004; Roth et al. 1989).

The temperature–mortality relationship for any given city is typically evaluated with time-series models that quantify the exposure of the entire population using near-surface temperatures measured at a single reference station, such as an airport (Basu 2009; Hajat et al. 2010). The most common indicators of heat exposure are air temperature and apparent temperature, where the latter combines measures of temperature and humidity (Basu 2009; Massetti et al. 2014; Steadman 1979). Either of these indicators can be quantified as the daily minimum, daily mean, or daily maximum, or as multi-day averages. Air temperature and apparent temperature are highly correlated (Basu 2009; Zhang et al. 2014), but apparent temperature has been more closely associated with mortality (Almeida et al. 2010; Barnett et al. 2010; Zhang et al. 2014). Furthermore, apparent temperature is a better predictor of indoor temperatures than air temperature (Nguyen et al. 2014), which is important for industrialized countries, where people spend > 90% of their time indoors (Höppe and Martinac 1998).

Although time-series studies using single reference station measurements are straightforward and methodologically consistent, they do not facilitate quantification of intra-urban spatial variability in the temperature–mortality relationship (Basu 2009). These spatial differences are likely driven by two overarching mechanisms: spatial variability in the exposure and spatial variability in the distribution of vulnerable populations. Previous efforts to evaluate the effect of spatial variability in exposure have typically characterized UHI impacts using satellite-derived estimates of land surface temperature (LST) (Hondula et al. 2012; Laaidi et al. 2012; Smargiassi et al. 2009). These studies have demonstrated that living in a relatively hot part of a city is a significant risk factor during extreme hot weather events, although the utility of LST for delineating these risks has been inconsistent (Ledrans et al. 2005). One possible reason for this inconsistency is that LST may not be the most appropriate proxy for human heat exposure, but it is often used because, unlike air temperature, it is directly measured by remote sensing instruments. Another possible reason is that many previous studies have used LST from the Moderate Resolution Imaging Spectroradiometer (MODIS), which has a coarse spatial resolution (1 km) (Laaidi et al. 2012; Tomlinson et al. 2011). Only a few studies have used more finely resolved data to describe spatial patterns of heat exposure (Hondula et al. 2012; Houghton et al. 2012; Johnson et al. 2012, 2014; Smargiassi et al. 2009; Uejio et al. 2011), and none has compared LST with temperature metrics that might better describe human health risks.

Individual and community risk factors that increase vulnerability to extreme hot weather have been the topic of studies in multiple contexts. In general, more vulnerable populations tend to be older, to have poorer physical and/or mental health, and to live in densely populated areas with high social and/or material deprivation (Kovats and Hajat 2008). Studies evaluating the spatial variability resulting from the distribution of vulnerable populations have typically focused on social risk factors, such as socioeconomic status (Buscail et al. 2012; Chow et al. 2012; Ho et al. 2015; Morabito et al. 2015; Reid et al. 2009; Tomlinson et al. 2011). For example, Reid et al. (2009) developed a qualitative index for mapping regional vulnerability to heat across the United States based on county-level indicators such as race, poverty, education, and social isolation. In further work, this vulnerability index was enhanced by including heat exposures as measured by LST, allowing isolation of areas assumed to be at the greatest risk during extreme hot weather (Buscail et al. 2012; Tomlinson et al. 2011). However, such indices apply universal measures of social and material deprivation that might not capture the place-specific nuances of risk within individual cities, such as the importance of access to transportation during the 1995 extreme hot weather event in Chicago (Semenza et al. 1996; Yardley et al. 2011). Furthermore, the performance of such vulnerability indices has not been rigorously evaluated with historical mortality data; this lack of rigorous evaluation has been flagged as a significant limitation of the work (Tomlinson et al. 2011). Even with excellent maps of exposure and vulnerability, it is important to consider that areas indicated to be at higher risk may not, in reality, experience excess deaths during extreme hot weather (O’Neill et al. 2009; Tomlinson et al. 2011).

The only way to accurately delineate areas of higher and lower risk is with data-driven methods designed to assess spatial differences in the temperature–mortality relationships across a city (Gabriel and Endlicher 2011; Greene et al. 2011; Gronlund et al. 2015; Hattis et al. 2012; Hondula et al. 2012, 2014, 2015; Petitti et al. 2016; Schaeffer et al. 2015; Schuster et al. 2014; Smargiassi et al. 2009). Herein, we report the development of an approach that combines data from a single representative weather station with exposure and vulnerability maps in a temporally and spatially stratified case-crossover design. This study is an extension of previous work conducted by Smargiassi et al. (2009) in Montreal, Canada. We applied these methods to greater Vancouver, Canada, where an extreme hot weather event during the summer of 2009 was associated with a 40% increase in mortality and > 100 excess deaths over a 7-day period (Henderson and Kosatsky 2012; Kosatsky et al. 2012). The specific objectives of our study were *a*) to assess spatial differences in the temperature–mortality relationship on extremely hot days using maps of LST, air temperature, and apparent temperature; *b*) to assess spatial differences in the temperature–mortality relationship on extremely hot days using maps of social vulnerability indicators; *c*) to evaluate whether areas at higher risk because of exposure and vulnerability have additive effects on the temperature–mortality relationship; and *d*) to map high-risk areas for emergency planning and public health protection during future extreme heat events.

## Methods

### Study Area

**Figure 1:**
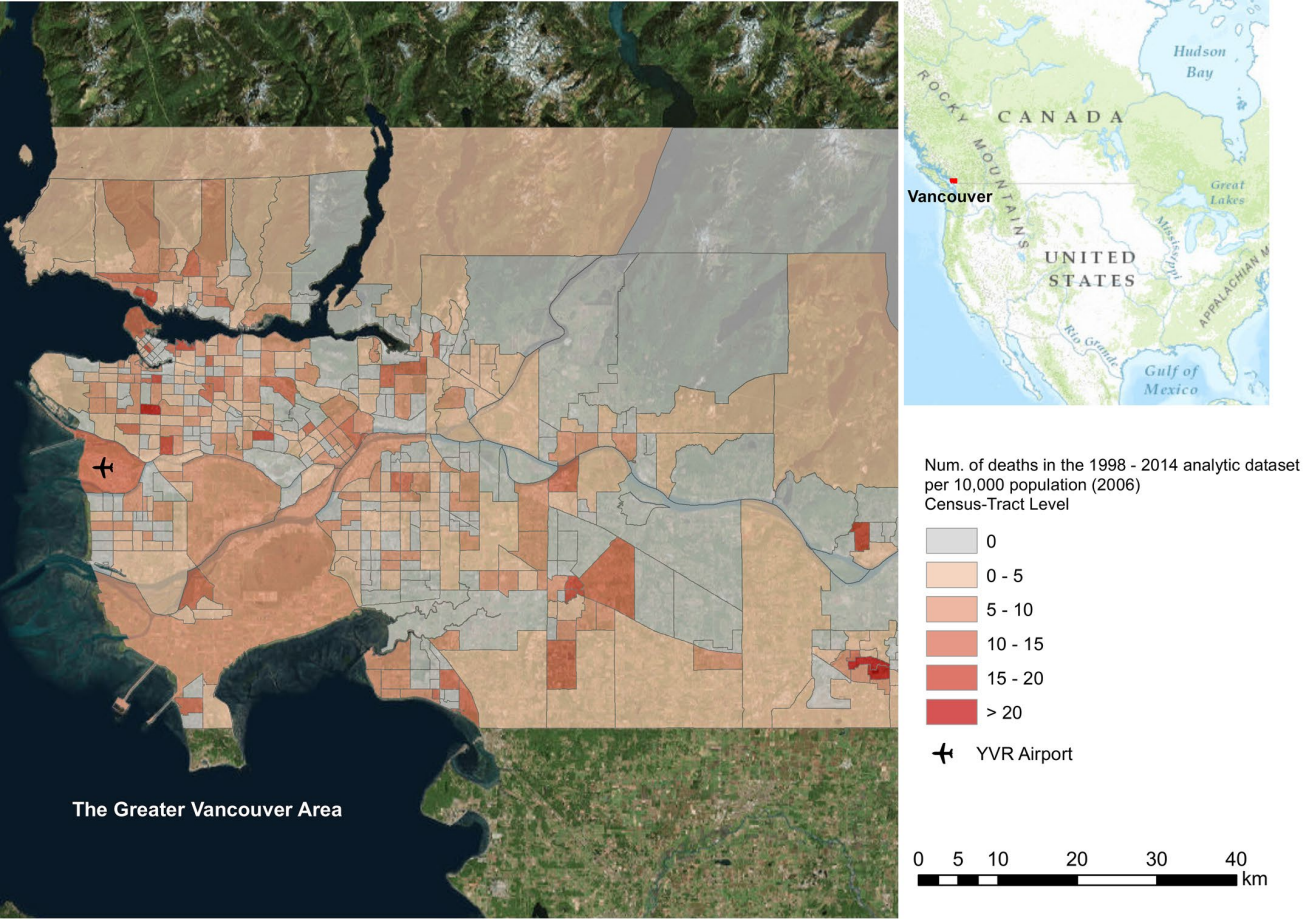
The greater Vancouver study area. The map indicates the number of deaths from each census tract that were included in the analytic data set over the entire study period. The base map of this map is the composite of imageries from ArcGIS 10.3.

The metropolitan area of greater Vancouver is on the west coast of British Columbia (BC), Canada (Figure 1), and has a population of > 2 million people. It is bordered by fold mountain ridges to the north, the Pacific Ocean to the west, and the semi-arid Fraser Valley to the east. This geographic context creates a complex mesoclimatic system that can lead to strong UHI effects on hot summer days (Oke 1976; Oke and Hay 1994; Runnalls and Oke 2000). During the summer, ocean breezes and winds from the mountain ridges can cool down the coastal regions, whereas the Fraser Valley can trap air masses and create a relatively hot zone (Oke and Hay 1994). For summer days (July and August) from 1998 through 2014, the average high, mean, and low air temperatures measured at Vancouver International Airport (YVR) were 22°C, 18°C, and 14°C, respectively. However, during the unprecedented hot weather event in 2009, daytime high temperatures reached 34°C and were > 30°C for 3 consecutive days. Although these temperatures are not hot by international standards, the 40% increase in mortality indicated that greater Vancouver was adversely affected by ambient temperatures that were high relative to seasonal norms (Kosatsky et al. 2012).

### Mortality Data

The BC Centre for Disease Control (BCCDC) receives daily data from the BC Vital Statistics Agency for the purposes of routine surveillance and evaluation of public health threats. We used data from 1998 through 2014 for our analyses. The database includes the following relevant information about each decedent: date of death, age in years, sex, underlying cause of death coded according to the World Health Organization’s *International Statistical Classification of Diseases and Related Health Problems* (10th Revision; ICD-10), and 6-digit residential postal code. An additional field describing the location of death (home, hospital, care facility, or other location) was added in 2008. All deaths resulting from transport accidents (ICD-10 codes V01–V99) were omitted, and the remaining deaths within greater Vancouver were geocoded by the residential 6-digit postal codes using the DMTI geodatabase (Statistics Canada 2007) with 99.99% success. We did not consider the underlying cause of death in our analytic framework because a previous study of the 2009 hot weather event showed no significant shifts when compared with typical summer weather (Kosatsky et al. 2012).

### Time-Varying Temperature Data

Hourly data from the Environment Canada weather station at YVR were used as the time-varying measure of exposure in the time-stratified case-crossover analyses. Every decedent was assigned daily mean humidex values for the day of death (case day) and for all occurrences of the same weekday within the same calendar month (control days). The humidex (Equation 1) is a measure of apparent temperature that is routinely used to quantify heat exposure in Canada (Burke et al. 2006; Gosling et al. 2014; Masterton and Richardson 1979).


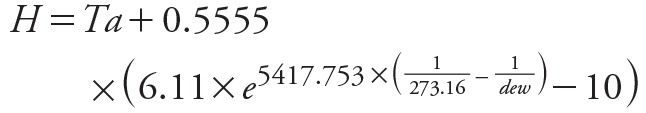
_[1]_

where *H* is the humidex; *Ta* is the air temperature; and *dew* is the dew point in Kelvin. Daily mean humidex was chosen as the time-varying temperature metric because previous work has shown that it is more strongly associated with mortality than air temperature in the study area (Henderson et al. 2013).

### Spatial Heat Exposure Data

To evaluate how the temperature–mortality relationship was associated with different measures of spatial variability in temperature, we used three previously developed heat exposure maps for LST, maximum daily air temperature, and maximum daily humidex. All three maps have a spatial resolution of 60 m and were derived from cloud-free Landsat images of greater Vancouver retrieved on hot summer days with maximum air temperatures ≥ 25°C at YVR (Table 1). The methods used to create each map are described in detail elsewhere (Barsi et al. 2003; Coll et al. 2010; Ho et al. 2014, 2016) and are described briefly below.

**Table 1:** Landsat images used for the creation of the land surface temperature, air temperature, and apparent temperature (humidex) maps.

Satellite platform	Acquisition date	Daily maximum air temperature at Vancouver International Airport (°C)
Landsat 5 TM	13 August 2002	27.1
Landsat 5 TM	7 July 2004	27.1
Landsat 7 ETM +	2 August 2004	25.4
Landsat 5 TM	23 July 2006	26.7
Landsat 5 TM	12 July 2008	25.5
Landsat 7 ETM +	31 July 2009	28.7

We derived LST from each of four Landsat 5 Thematic Mapper (TM) images (Table 1) using standard methods (Barsi et al. 2003; Coll et al. 2010) and took the average of the four values in every pixel to create a single map of LST for a typical hot summer day (see Figure S1). The same four Landsat 5 TM images and two Landsat 7 Enhanced Thematic Mapper (ETM+) images (Table 1) were used to create the air temperature map (Ho et al. 2014). A nonlinear random forest model was used to estimate the relationship between several spatial variables and maximum air temperature at 59 local weather stations on the days of the Landsat images. Random forest is a nonparametric machine learning approach that uses an ensemble of regression trees to model the relationship between a response variable and multiple predictors. Each tree is trained on a random subset of the training data, with a random subset of the available predictors used to split the data in each node of each tree (Breiman 2001). The spatial predictors used to train the air temperature model were LST, normalized difference water index (Gao 1996), skyview factor derived from local LIDAR data and Landsat 5 TM, and elevation and solar radiation (Fu and Rich 2002) derived from the Geobase digital elevation model (CDED, http://www.geobase.ca). The skyview factor describes the percentage of unobscured sky, which is related to the radiation received or emitted in an area (Chen et al. 2012; Ho et al. 2014; Su et al. 2008). We applied the random forest model to create a single map of the maximum estimated air temperature (see Figure S2). The mean absolute error between the observed and predicted air temperatures was 1.82°C. A similar approach was used for the humidex map (see Figure S3), with a mean absolute error of 1.67°C (Ho et al. 2016). These errors are small compared with those in similar studies in the remote sensing literature (Benali et al. 2012; Ho et al. 2014; Xu et al. 2014).

### Spatial Social Vulnerability Data

Deprivation indices are commonly used for describing social and health disparity across Canada (Bell et al. 2007; Bell and Hayes 2012; Pampalon et al. 2009, 2012; Walker et al. 2014). The Vancouver Area Neighborhood Deprivation Index (VANDIX) is a combined measure of material and social deprivation that was developed specifically for health research in the study area. Researchers asked 27 local medical health officers to rank 21 census variables with respect to their perceived importance to public health in the region, and 10 officers returned the completed survey (Bell et al. 2007; Bell and Hayes 2012). Of the 21 variables, 7 were used to construct the VANDIX in ranked order of importance: percent of the population that did not finish high school, unemployment rate, percent of the population with a university education, percent of single-parent families, average income, percentage of homes owned, and labor participation rate. The last variable refers to the percent of the adult population that is either employed or actively looking for work.

We used the VANDIX itself (see Figure S4) and all seven contributing variables (see Figures S5–S11) to evaluate spatial differences in the temperature–mortality relationship related to social vulnerability. In addition, we considered some census variables used for the common heat vulnerability indices (Buscail et al. 2012; Morabito et al. 2015; Reid et al. 2009; Tomlinson et al. 2011). These included the density of the population aged ≥ 55 (see Figure S12), the density of persons living alone (see Figure S13), and the density of housing built before 1970 (see Figure S14). The values for all variables and the resulting VANDIX were taken from the 2006 national census using the SimplyMap 3.0 database (http://geographicresearch.com/simplymap/). The mandatory 2006 census was selected because it was conducted in the middle of the 1998–2014 study period and because the voluntary 2011 census resulted in significantly lower data quality than its 2006 predecessor (Coleman 2013; Green and Milligan 2010). The data were mapped at the level of census dissemination area, where each spatial unit contains an approximate population of 400–700 persons (Statistics Canada 2007).

There were four variables for which increasing risk was assumed to be associated with decreasing values: percent of the population with a university education, average income, percent of homes owned, and labor participation rate. To ensure consistency of interpretation between variables, we expressed these as percent of the population without a university degree, average income ratio, percent of homes rented, and the labor nonparticipation rate, respectively. The average income ratio for each dissemination area was calculated as the minimum average income for all dissemination areas divided by the average income for that area.

### Spatial Delineation of the Temperature–Mortality Relationship using Case-Crossover Analyses

The residential location of every successfully geocoded record in the mortality data set was assigned a single value from each of the heat exposure and social vulnerability maps. We considered these 14 variables to be potentially indicative of spatial differences in the temperature–mortality relationship as assessed with time-varying mean humidex measured at the fixed YVR reference site. Given our interest in spatial variation during extreme hot weather, the mortality data set was then reduced to all records with a case day or a control day in the 99.9th percentile (≥ 24°C) of daily mean air temperature values measured at YVR from 1998 through 2014. In other words, the analytic data set included all deaths that occurred on extremely hot days and all deaths that occurred on comparable nonextreme days. Conditional logistic regression was used to estimate the odds ratio (OR) for mortality associated with a 1°C increase in daily mean humidex at YVR. All analyses were conducted in R with the *survival* package (Therneau 2015).

**Figure 2:**
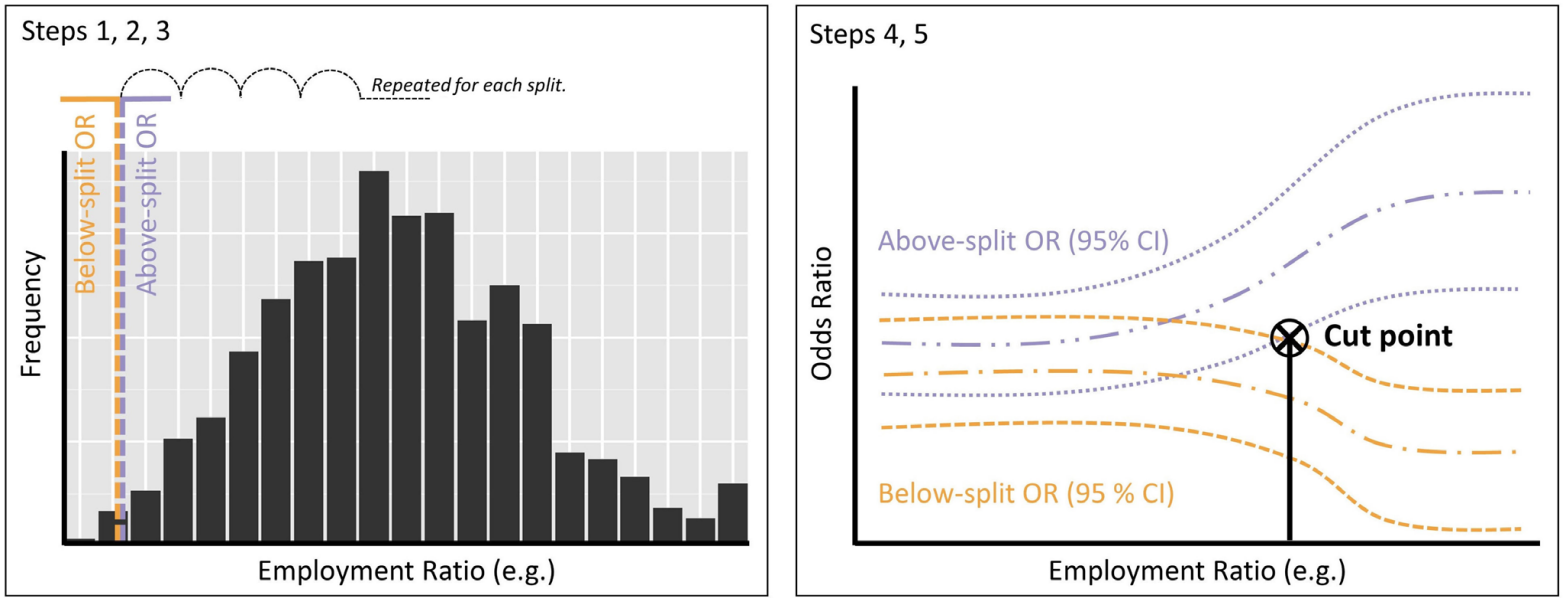
Illustration of the methods used for the spatial delineation. The left side shows how the split values were defined, and the right side shows how the cut point was identified.

The spatial delineation was conducted for each of the 14 spatial variables as follows: *a*) We took the values of the variable for all decedents and placed splits at the 1st and 99th percentiles; *b*) we placed another 98 splits at equally spaced intervals (not percentiles) between these values; *c*) for each of the 100 splits, a new binary variable was assigned to each decedent, with those who lived in areas below the split receiving a value of 0 and those who lived in areas equal to or greater than the split receiving a value of 1; *d*) the conditional logistic regression model was run with a term for interaction between time-varying mean humidex and the new dichotomous variable (Equation 2), producing ORs and 95% confidence intervals (CIs) for areas above and below the splits; and *e*) we plotted the results for the below-split and above-split results as two smooth lines in one graph. Where there is increasing risk of mortality associated with increasing values of a spatial variable, we expect the above-split ORs to increase as the split value increases. For variables where there was a positive relationship with the above-split ORs, we identified a single cut point to spatially delineate areas of higher risk based on the first separation of the below-split and above-split confidence intervals (Figure 2), and we calculated the slope of the positive relationship above the cut point.

logit(*Death*|*UID*) = β_0_ + β_1_ *H* × *Above Split*, [2]

where *Death* is 1 on case days and 0 on control days, conditioned on the unique identifier (*UID*) of each decedent; *H* is the daily mean humidex measured at YVR; and *Above Split* is the binary variable indicating whether the residence of the decedent was above or below the split value.

### Combined Heat Exposure and Social Vulnerability Index

One motivation for spatial delineation of the temperature–mortality relationship is to map areas of potential high risk for future environmental health assessment and emergency planning (Reid et al. 2009; Tomlinson et al. 2011). Based on results from the individual variables, we wanted to evaluate whether the temperature–mortality relationship was further elevated in areas with combined risks from heat exposure and social vulnerability. To build a composite index, the strongest temperature and social vulnerability variables were first reclassified to percentile maps using their high-risk cut points and their minimum/maximum values from the analytic data set (Brewer and Pickle 2002; Erhardt et al. 2012). Locations lower than the cut points were reclassified from 0% to 50% by taking percentiles from the minimum value to the cut point value such that each bin contained 0.5% of the data. The same method was applied to the locations above the cut points, where the variables were reclassified from 50% to 100%. The composite index was then calculated as the average of the two reclassified variables (Equation 3).

*Composite Index* = 0.5 * *Temperature*_re_ + 0.5 * *SocialVulnerability*_re_, [3]

where *Temperature*_re_ is the reclassified temperature variable and *SocialVulnerability*_re_ is the reclassified social vulnerability variable.

### Sensitivity Analysis

This study used administrative mortality data from the BC Vital Statistics Agency, and values of the spatial variables were assigned to decedents using their residential postal codes, regardless of where they were exposed or died. It is likely that some who died in hospital were not exposed at their residential locations on the day of death, meaning that they were spatially misclassified, particularly for the heat exposure variables. To assess the potential impacts of this misclassification, we restricted the analytic data set to the subset of decedents who were known to have died out-of-hospital and repeated all analyses to evaluate the impacts.

## Results

### Data Summary

The analytic data set included cases who died on extremely hot days (mean air temperature ≥ 24°C at YVR) and cases who died on cooler days that had an extremely hot day as a control day. There were 6 extremely hot days during the 1998–2014 study period (2 in 1998, 1 in 2006, and 3 in 2009), and 267 deaths occurred on those dates. There were 21 cooler days that had an extremely hot day as a control day, and 730 deaths occurred on those dates (Table 2). The mean humidex at YVR on the extremely hot days was 25.6°C (standard deviation: 1.6°C), and the mean of the maximum values was 31.2°C (standard deviation: 2.1°C). The number of deaths on extremely hot days ranged from 26 to 65, with the highest number of deaths occurring on the hottest day. The number of deaths on the cooler days ranged from 15 to 47 (Table 2). Cases who died on extremely hot days had consistently higher heat exposures and indicators of social vulnerability, but differences between groups were significant for only three variables: VANDIX, average income ratio, and percent of homes rented (Table 2).

**Table 2:** Summary statistics for the analytic data set. Summaries are presented for cases who *a*) died on the extremely hot days [mean air temperature ≥ 24°C at Vancouver International Airport (YVR)] and *b*) who died on a cooler day that had one of the extremely hot days as a control day.

Summary statistic	Cases who died on 6 extremely hot days	Cases who died on 21 cooler days with an extremely hot day as a control day
Total	267	730
Average (range) deaths per day	44.5 (26–65)	34.1 (15–47)
Percent male	50.2	48.6
Mean (SD) age at death (years)	75.5 (15.7)	74.7 (18.6)
Mean (SD) land surface temperature (°C)	40.0 (3.1)	38.8 (3.2)
Mean (SD) air temperature (°C)	30.1 (0.87)	30.0 (0.86)
Mean (SD) humidex (°C)	32.9 (1.4)	32.7 (1.4)
Mean (SD) VANDIX	**0.02 (0.67)**	**–0.09 (0.60)**
Mean (SD) percent not graduated from high school	19.6 (11.3)	18.8 (10.0)
Mean (SD) unemployment rate (%)	6.3 (4.2)	5.8 (3.9)
Mean (SD) percent with no university degree	77.9 (14.0)	77.3 (12.9)
Mean (SD) percent of single-parent families	16.3 (10.6)	15.3 (8.6)
Mean (SD) average income ratio	**37.4 (13.5)**	**34.8 (11.4)**
Mean (SD) percent of homes rented	**37.7 (27.4)**	**33.9 (25.6)**
Mean (SD) labor nonparticipation rate	38.1 (15.7)	36.7 (13.0)
Mean (SD) density of population ≥ 55 years (per km^2^)	1,627 (2,056)	1,551 (1,661)
Mean (SD) density of persons living alone (per km^2^)	1,145 (2,269)	1,070 (2,311)
Mean (SD) density of housing built before 1970 (per km^2^)	831 (1,630)	828 (1,803)
Notes: VANDIX, Vancouver Area Neighborhood Deprivation Index. Values in bold indicate a statistically significant difference between means (*t*-test with alpha = 0.05)		

### Spatial Variability in the Temperature–Mortality Relationship by Heat Exposure

**Figure 3:**
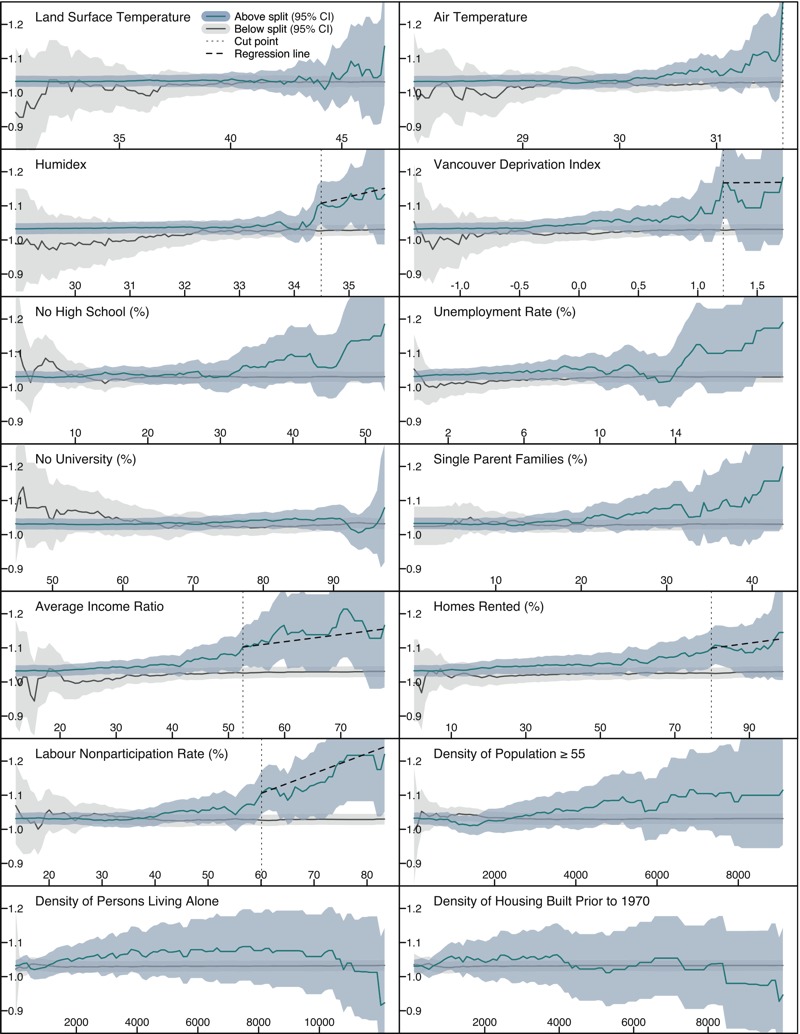
Each figure indicates the odds of mortality associated with a 1°C increase in daily mean humidex (apparent temperature) for decedents who lived in areas above (blue lines) and below (gray lines) each split value on the *x*-axis. The cut point is marked with a vertical dashed line where applicable, indicating the first statistical separation between 95% confidence intervals. The slope of the above-split relationship after the cut point is shown as a solid black line where applicable. The population and dwelling densities are in units per square kilometer.

There were spatial differences in risk between the LST, air temperature, and humidex maps (Figure 3). For LST, there was near-complete overlap of the confidence intervals for ORs below and above all split values. For air temperature, there was some statistically nonsignificant separation of the lines starting at values ~29.8°C, with the only significant difference observed in areas > 31.7°C. For humidex, there was a sharp increase in risk above the split value for areas > 34.2°C, with the first significant difference observed at 34.4°C. At this value, the odds ratio (OR) for a 1°C increase in daily mean humidex at YVR was 1.09 [95% CI: 1.04, 1.15] for data above the cut point, compared with 1.03 [95% CI: 1.01, 1.04] for data below the cut point. The area above the cut point included 12.0% of deaths on extremely hot days and 6.7% of deaths on the cooler days.

### Spatial Variability in the Temperature–Mortality Relationship by Social Vulnerability

We used 11 variables to assess spatial differences in the temperature–mortality relationship associated with social vulnerability. Of these, only 4 showed statistically significant separation of the odds ratios above and below any of the split values (Figure 3). These variables were VANDIX (≥ 1.2), average income ratio (≥ 52%), percent of homes rented (≥ 80%), and the labor nonparticipation rate (≥ 60%). The slope of the relationship above the cut point was flat for VANDIX, and the strongest positive relationship was for the labor nonparticipation rate. Above the cut point value of 60%, the OR for a 1°C increase in daily mean humidex at YVR was 1.11 [95% CI: 1.04, 1.17], compared with 1.03 [95% CI: 1.01, 1.04] below the cut point value. The area above this value included 9.4% of deaths on extremely hot days and 5.2% of deaths on the cooler days. If the cut point value was moved to 75%, the OR for a 1°C increase in daily mean humidex at YVR was 1.20 [95% CI: 1.07, 1.34]. The area above this value included 3.7% of deaths on extremely hot days and 1.0% of deaths on the cooler days.

### Sensitivity Analysis with Out-of-Hospital Deaths

To assess the potential impacts of spatial misclassification in the exposure assessment, we restricted the analytic data set to the 25.8% subset of 257 decedents who were known to have died out-of-hospital. Of these, 160 died in a residential institution, and 97 died at home. Those with values of ≥ 34.4°C on the spatial humidex map had an OR of 1.15 [95% CI: 1.06, 1.25], compared with 1.09 [95% CI: 1.04, 1.15] for the entire analytic data set. Similarly, those living in areas where the labor nonparticipation rate was ≥ 60% had an OR of 1.22 [95% CI: 1.11, 1.34], compared with 1.11 [95% CI: 1.04, 1.17] for the entire analytic data set.

### Combined Heat Exposure and Social Vulnerability Index

**Figure 4:**
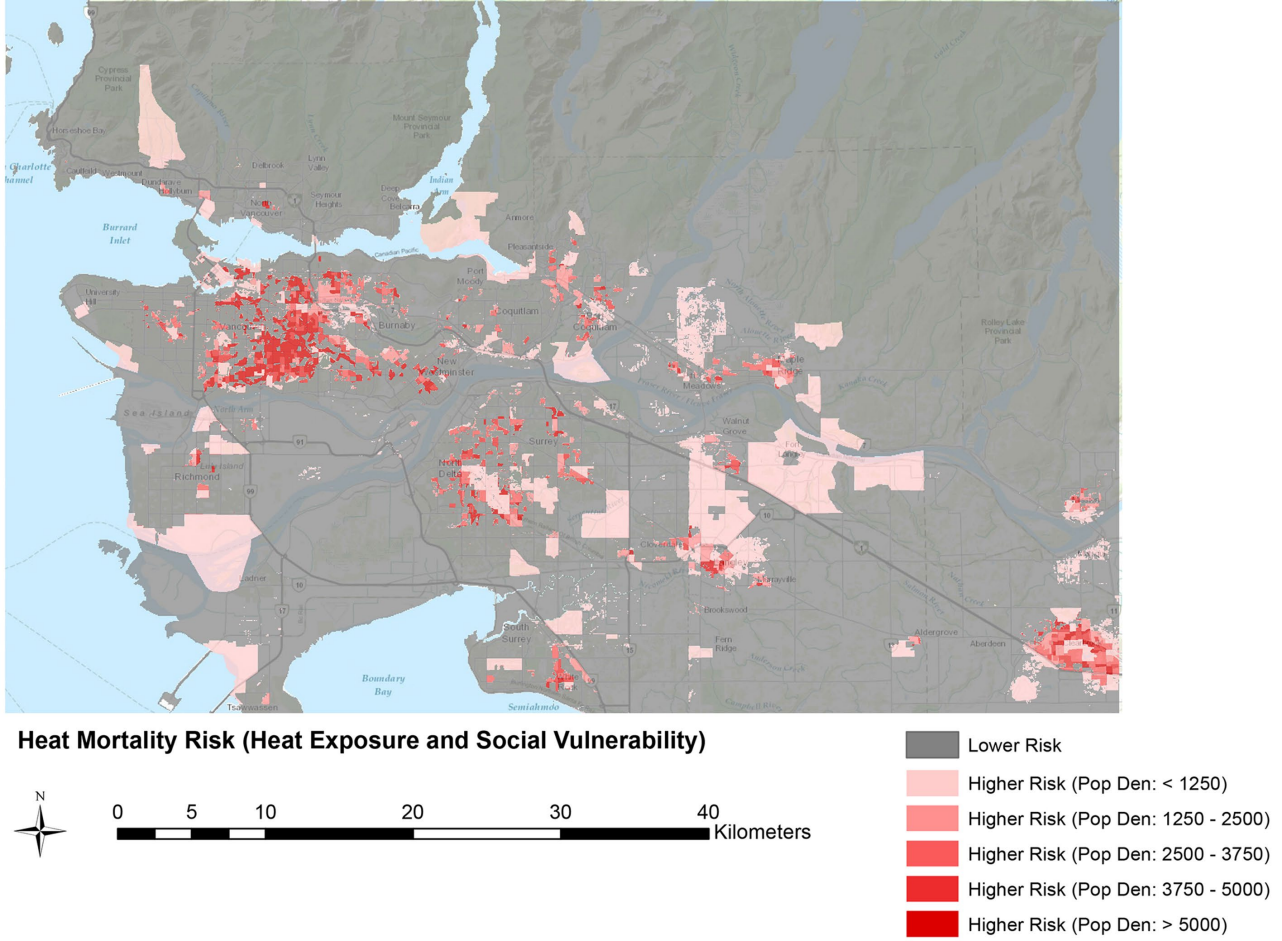
Spatial delineation of the composite index using the out-of-hospital mortality subset. Values of the index greater than 37% are classified as higher risk and are shown here by increasing population density (per square kilometer) in order to display the relationship between higher risk and population distribution in the greater Vancouver study area. The base map of this map is the topographic map from ArcGIS 10.3.

**Figure 5:**
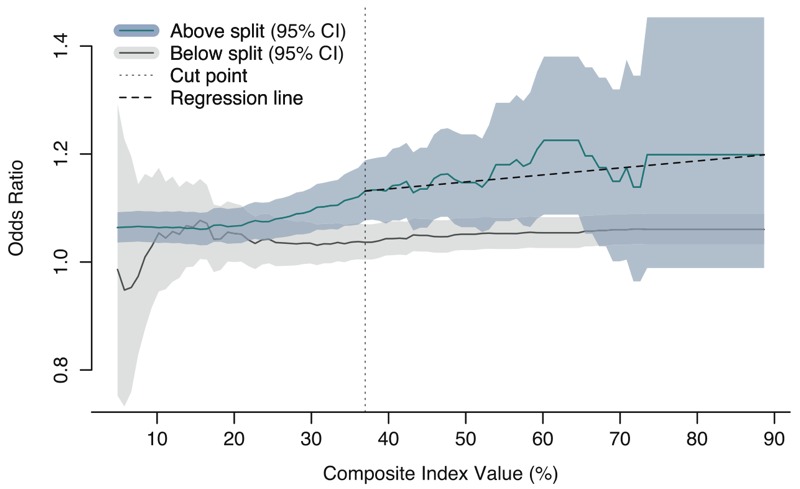
The odds of mortality associated with a 1°C increase in daily mean humidex (apparent temperature) for out-of-hospital decedents who lived in areas above (blue lines) and below (gray lines) each split value of the composite index. The cut point is marked with a vertical dashed line, indicating the first statistical separation between 95% confidence intervals. The slope of the above-split relationship after the cut point is shown as a solid black line.

Maps of the areas above and below the humidex and labor nonparticipation cut points (34.4°C and 60%, respectively) indicated little spatial overlap. The humidex map showed that urban and suburban residential areas were at higher heat risk, whereas the labor nonparticipation map highlighted different areas of high deprivation, including the downtown east side, one of the most socioeconomically deprived neighborhoods in Canada (Kerr et al. 2003). Although extreme values of these two variables are spatially disparate for greater Vancouver, there could be interaction between them at lower values to result in an elevated risk. To better quantify the heat risk, we created a map of the composite index to visualize high-risk areas for use in emergency planning (Figure 4). The below- and above-split analyses showed no statistical separation of ORs above and below any split value, but the point of maximum separation of the confidence intervals was at 46%. However, when the data set was restricted to out-of-hospital deaths only, there was significant separation of the confidence intervals at 37%, followed by a weakly positive association (Figure 5). At this value, the OR for a 1°C increase in daily mean humidex at YVR was 1.13 [95% CI: 1.08, 1.19] for data above the cut point, compared with 1.04 [95% CI: 1.00, 1.07] for data below the cut point.

## Discussion

We have used data from extremely hot days to assess spatial variability in the temperature–mortality relationship associated with differences in heat exposure and social vulnerability in greater Vancouver, Canada. We found that maps of the humidex and labor nonparticipation rates showed the strongest spatial differences in this study area, and we developed a combined index of these variables to further delineate areas of increased risk. The analytic approach used a time-stratified case-crossover design to examine differences between the ORs for cases with residential locations above and below a complete range of split values for each spatial variable. Where complete separation of the ORs above and below a cut point was observed, the odds of mortality for those above the cut point was increased by 10–11% for each 1°C increase in daily mean humidex at YVR, compared with 2–3% for those below the cut point.

These results are somewhat consistent with those of other, comparable studies (Gronlund et al. 2015; Hondula et al. 2015; Smargiassi et al. 2009). For example, Hondula et al. (2015) used baseline mortality at 10°C and observed mortality on hot days to report a 0–10% increase in risk for areas with temperatures > 30°C in multiple U.S. cities. In comparison, locations between 20°C and 30°C had increases in risk ranging from –2.5% to 2.5%. In another spatially stratified case-crossover analysis of social vulnerability, Gronlund et al. (2015) reported no significant differences in ORs for cardiovascular or respiratory mortality between all cases and those cases that occurred in areas of Michigan presumed to be vulnerable. The one exception was for respiratory mortality in areas with older housing. Finally, Smargiassi et al. (2009) reported a 6–7% increase in odds of nonaccidental mortality for areas of Montreal, Canada, where LST was used to identify areas > 2°C hotter than the fixed reference site.

One strength of our study was its comparison between LST, air temperature, and humidex for mapping differences in the temperature–mortality relationship, where others have used LST without further evaluating its relative suitability for this purpose. Although we observed a small increase in the OR for increasing LST values, it dropped rapidly, and no separation of ORs was observed at any split value. These observations might suggest that LST is not an appropriate metric for this particular study area, but further evaluation of LST for meaningful delineation of the temperature–mortality relationship is needed in all contexts. In comparison, we found that our previously developed maps of maximum air temperature and humidex on hot days (Ho et al. 2014, 2016) showed significant increases in the temperature–mortality relationship as the temperatures increased, with the stronger relationship observed for the humidex map. Our methods indicated a statistically significant difference in risk for those living in areas ≥ 34.4°C, which includes ~7% of the greater Vancouver population.

Another strength of this study was its data-driven comparison of several variables reflecting different social vulnerabilities with the potential to be associated with increased risk of mortality during extreme heat. Of the 11 variables evaluated, only 4 showed significantly increased risk in some areas: VANDIX (which did not show a positive relationship after the cut point), average income ratio, percent of homes rented, and labor nonparticipation rate. The last 3 variables all contribute to the modified composite VANDIX, along with 4 variables for which no significantly increased risks were found: percent with no high school, unemployment rate, percent with no university, and percent single-parent families. Although it is valuable to know that the VANDIX does delineate risk in the greater Vancouver area for which it was designed, it is also valuable to know that not all of the contributing variables can adequately capture the heat-health risks associated with deprivation. Furthermore, no areas of significantly increased risk were observed for variables reflecting the densities of the senior population, of persons living alone, and of housing built before 1970 in greater Vancouver. These are important observations because some spatial indices of hot-weather vulnerability use such variables based on a general association that may not, in fact, exist in specific areas to which they are applied (Reid et al. 2009; Yardley et al. 2011). Here, we report that labor nonparticipation was the strongest indicator of social vulnerability for greater Vancouver, but this finding may not hold in other cities with different geographic and demographic contexts.

The methods we have presented here use spatially stratified case-crossover analyses to statistically delineate areas of higher and lower mortality risk on extremely hot days. This approach is limited by its strict separation of contiguous areas that might actually be at similar risk. It would be valuable for future work to develop methods that account for such data clustering while maintaining adequate power for the analyses. We did experiment with binning data by variable ranges that included 20%, 10%, 5%, and 2% of the analytic data set, but we found that the larger ranges did not produce spatially stratified results and that the smaller ranges did not achieve statistical significance. Other approaches may include moving windows or fuzzy boundaries that include all deaths within some tolerance of spatial values of the strictly delineated areas. Such advances may also help to improve maps of composite risk from combined heat exposure and social vulnerability, thereby improving environmental health planning for future events (Tomlinson et al. 2011).

Another limitation of our study was the definition of the analytic data set using daily mean air temperature ≥ 24°C at YVR. Given our objective to assess the mortality risks on extremely hot days, we hypothesized that including data from lower temperatures would serve to attenuate the spatially stratified results we were hoping to highlight. To test this hypothesis, we repeated all analyses using values of 20–26°C to create the analytic data set. When results for the humidex map and the nonparticipation rate were compared with those reported here, we found that similar spatially stratified results were evident at the ≥ 20°C restriction but that statistical separation was not evident until the ≥ 24°C restriction. This separation persisted for the humidex map at the ≥ 25°C and ≥ 26°C restrictions, despite the reduced power of the smaller data sets. However, for the purposes of heat-risk mapping, it might be informative to work with a larger analytic data set restricted by some other variable. For example, our sensitivity analyses with the 25.8% subset of cases who died out-of-hospital appeared more sensitive to spatial differences than the entire subset defined by temperature. Given that extreme hot weather is associated with increased incidence of out-of-hospital deaths (Kosatsky et al. 2012; O’Neill et al. 2003), we recommend that future work focuses on this subset of decedents to most effectively delineate areas of risk.

## Conclusion

We have presented a novel application of the time-stratified case-crossover study design for spatial delineation of differences in the temperature–mortality relationship on extremely hot days. We have also evaluated the utility and appropriateness of different heat exposure maps and measures of social vulnerability. The results showed that humidex and labor nonparticipation were most strongly associated with spatial differences in the temperature–mortality relationship for greater Vancouver and that there was no association for most variables. A composite index showed some areas where combined heat exposure and social vulnerability led to increased risk. Our study highlights the need for data-driven mapping of the temperature–mortality relationship to best prepare individual cities for public health protection during extreme hot weather events.

## Supplemental Material

(1.7 MB) PDF
