# Supplementary material for: Delineation of Spatial Variability in the Temperature–Mortality Relationship on Extremely Hot Days in Greater Vancouver, Canada
Source: Environ Health Perspect. 2016 Jun 27;125(1):66–75. doi: 10.1289/EHP224 (PMC5226699; doi:10.1289/EHP224)
Supplement: (1.7 MB) PDF [file EHP224.s001.acco.pdf]

Environ Health Perspect
DOI: 10.1289/EHP224

Note to readers with disabilities: *EHP* strives to ensure that all journal content is accessible to all readers. However, some figures and Supplemental Material published in *EHP* articles may not conform to 508 standards due to the complexity of the information being presented. If you need assistance accessing journal content, please contact ehp508@niehs.nih.gov. Our staff will work with you to assess and meet your accessibility needs within 3 working days.

Supplemental Material

Delineation of Spatial Variability in the Temperature-Mortality Relationship on Extremely Hot Days in Greater Vancouver, Canada

Hung Chak Ho, Anders Knudby, Blake Byron Walker, and Sarah B. Henderson

Table of Contents

Figure S1. Map of the Land Surface Temperature (°C) spatial variable 2
Figure S2. Map of the Air Temperature (°C) spatial variable 3
Figure S3. Map of the Humidex (°C) spatial variable 4
Figure S4. Map of the Vancouver Deprivation Index (VANDIX) spatial variable 5
Figure S5. Map of the No High School (%) spatial variable 6
Figure S6. Map of the Unemployment Rate (%) spatial variable 7
Figure S7. Map of the No University (%) spatial variable 8
Figure S8. Map of the Single Parent Families (%) spatial variable 9
Figure S9. Map of the Average Income Ratio spatial variable 10
Figure S10. Map of the Homes Rented (%) spatial variable 11
Figure S11. Map of the Labour Nonparticipation Rate (%) spatial variable 12
Figure S12. Map of the Density of Population ≥ 55 (km^{-2}) spatial variable 13
Figure S13. Map of the Density of Persons Living Alone (km^{-2}) spatial variable 14
Figure S14. Map of the Density of Housing Built Prior to 1970 (km^{-2}) spatial variable 15
References 16

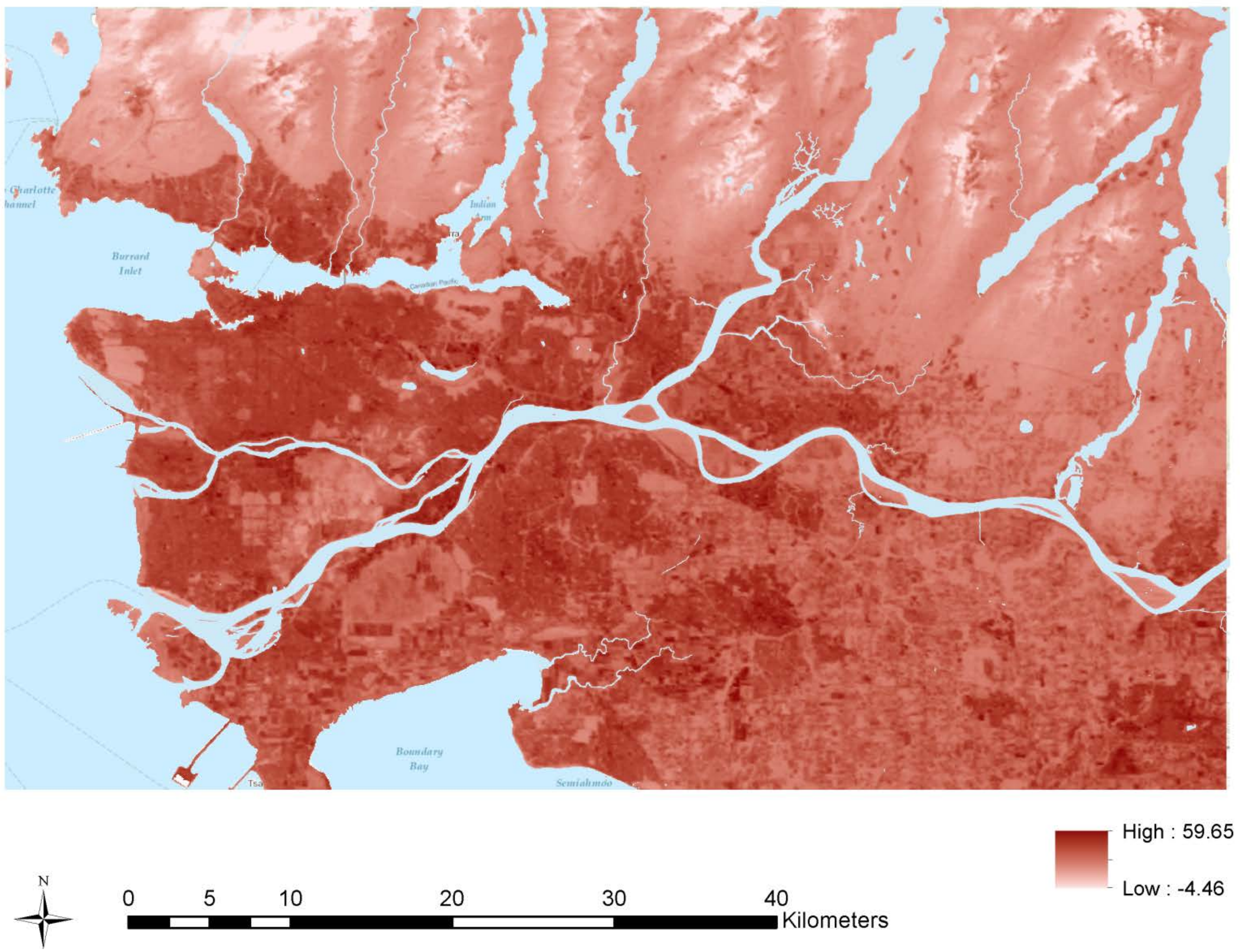

Figure S1. Map of the Land Surface Temperature (°C) spatial variable

Map was generated by averaging data from Landsat on six hot days in greater Vancouver, as described briefly in the main text and in detail elsewhere (Ho et al. 2014; Ho et al. 2016).

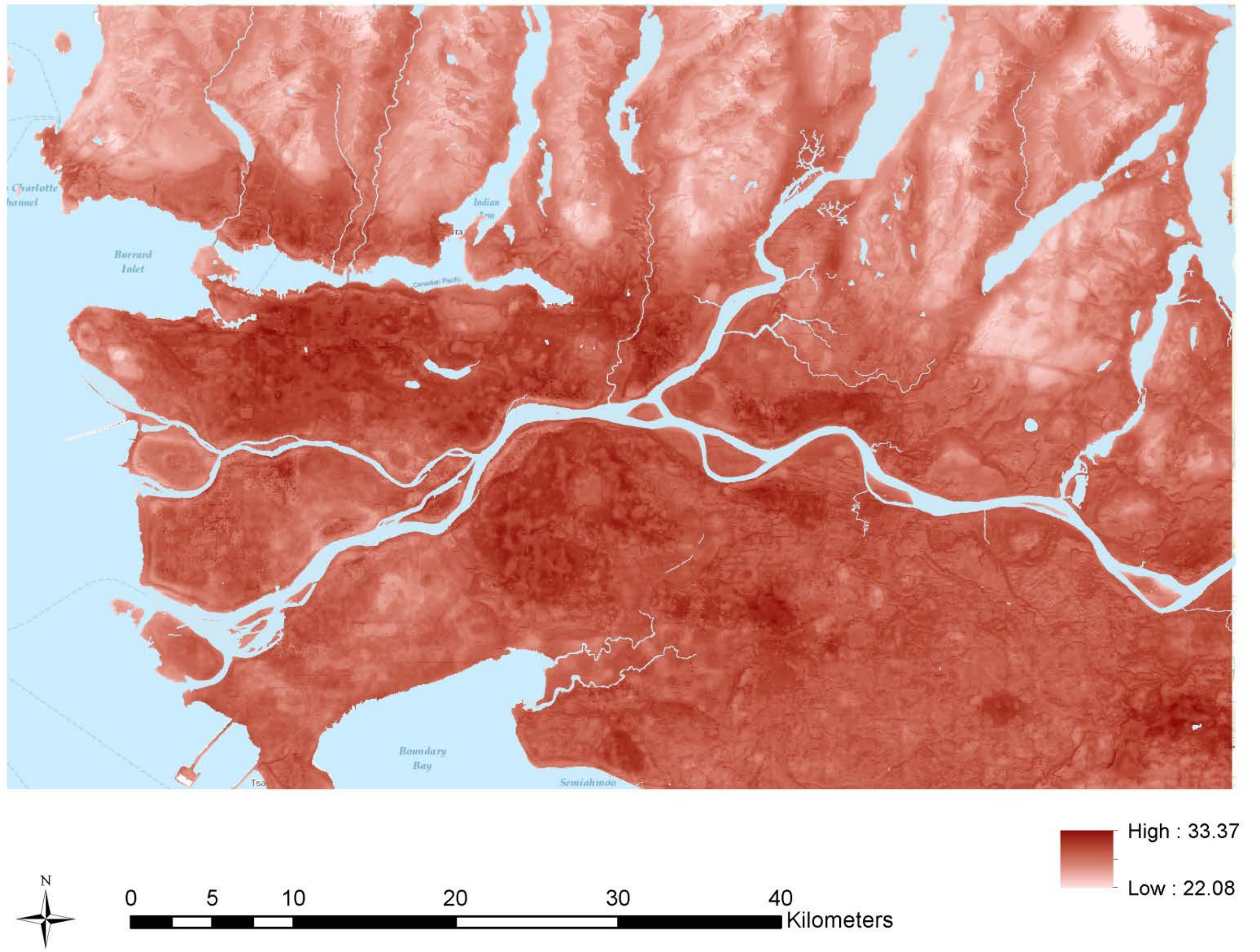

Figure S2. Map of the Air Temperature (°C) spatial variable

Air temperature was estimated according to methods published elsewhere (Ho et al. 2014) and briefly described in the main text.

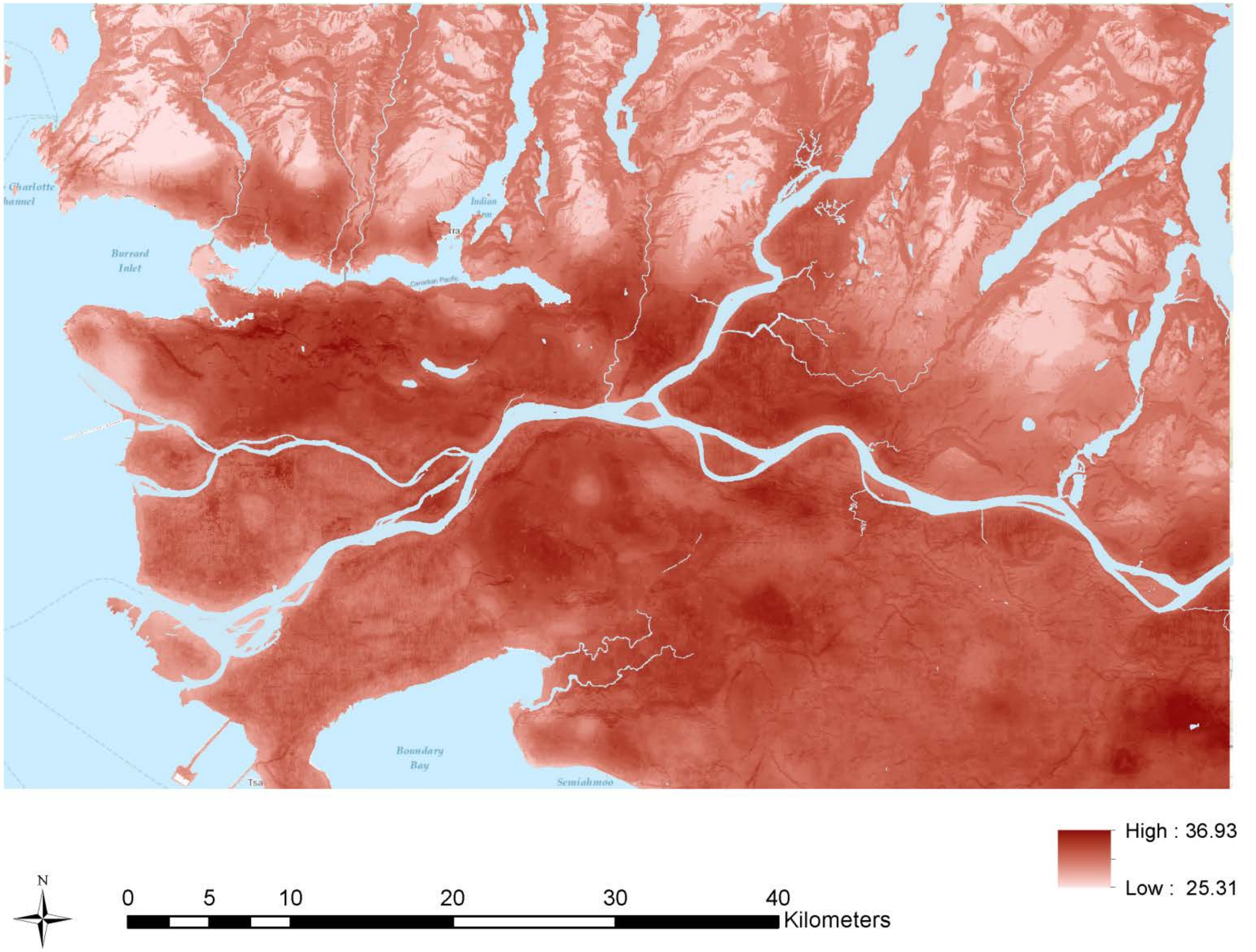

Figure S3. Map of the Humidex (°C) spatial variable

Humidex was estimated according to methods published elsewhere (Ho et al. 2016) and briefly described in the main text.

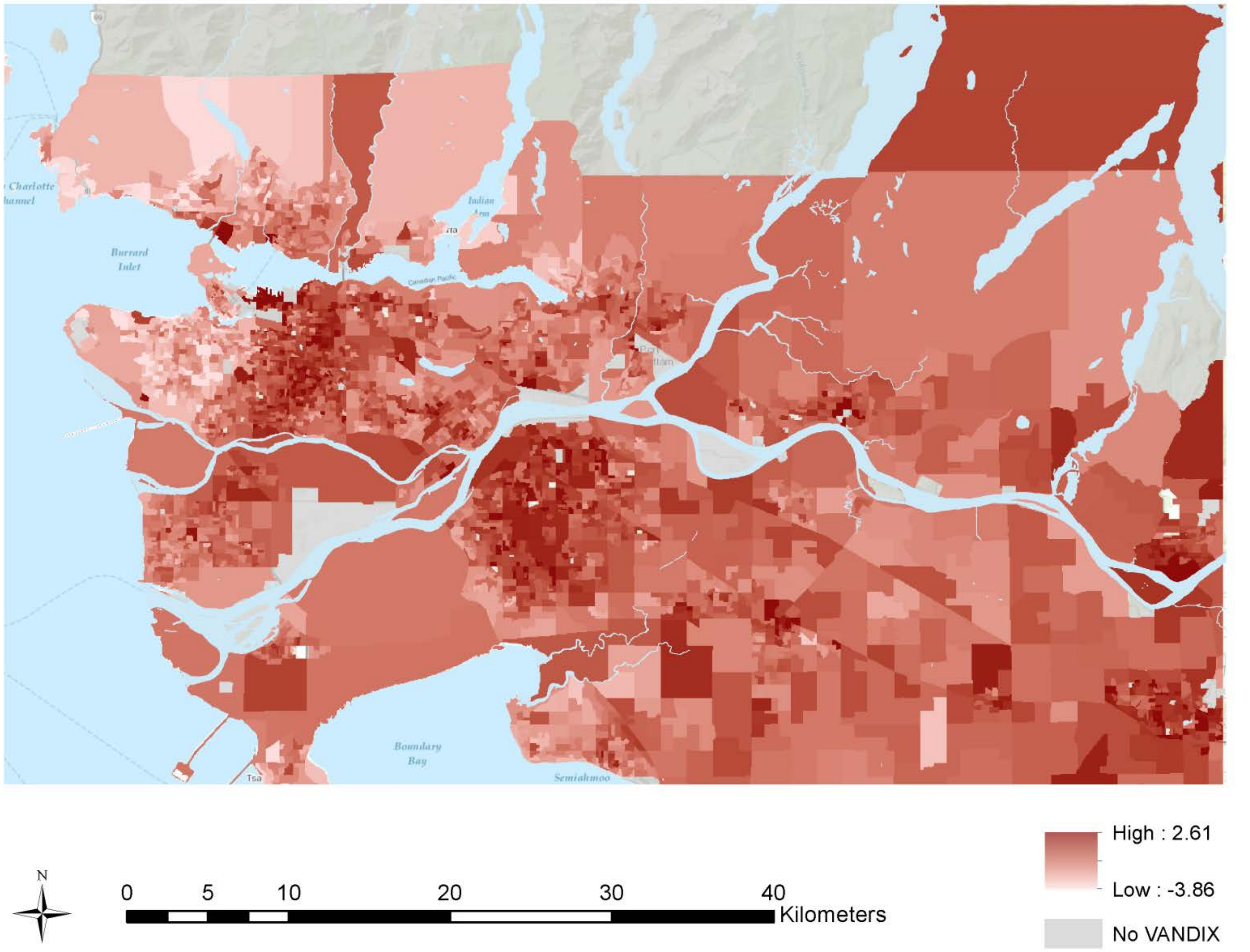

Figure S4. Map of the Vancouver Deprivation Index (VANDIX) spatial variable

The VANDIX was mapped using data from the 2006 national census, sourced from SimplyMap (Geographic Research Inc 2015) and calculated according to methods described briefly in the main text and in detail elsewhere (Bell et al. 2007; Bell and Hayes 2012; Walker et al. 2014).

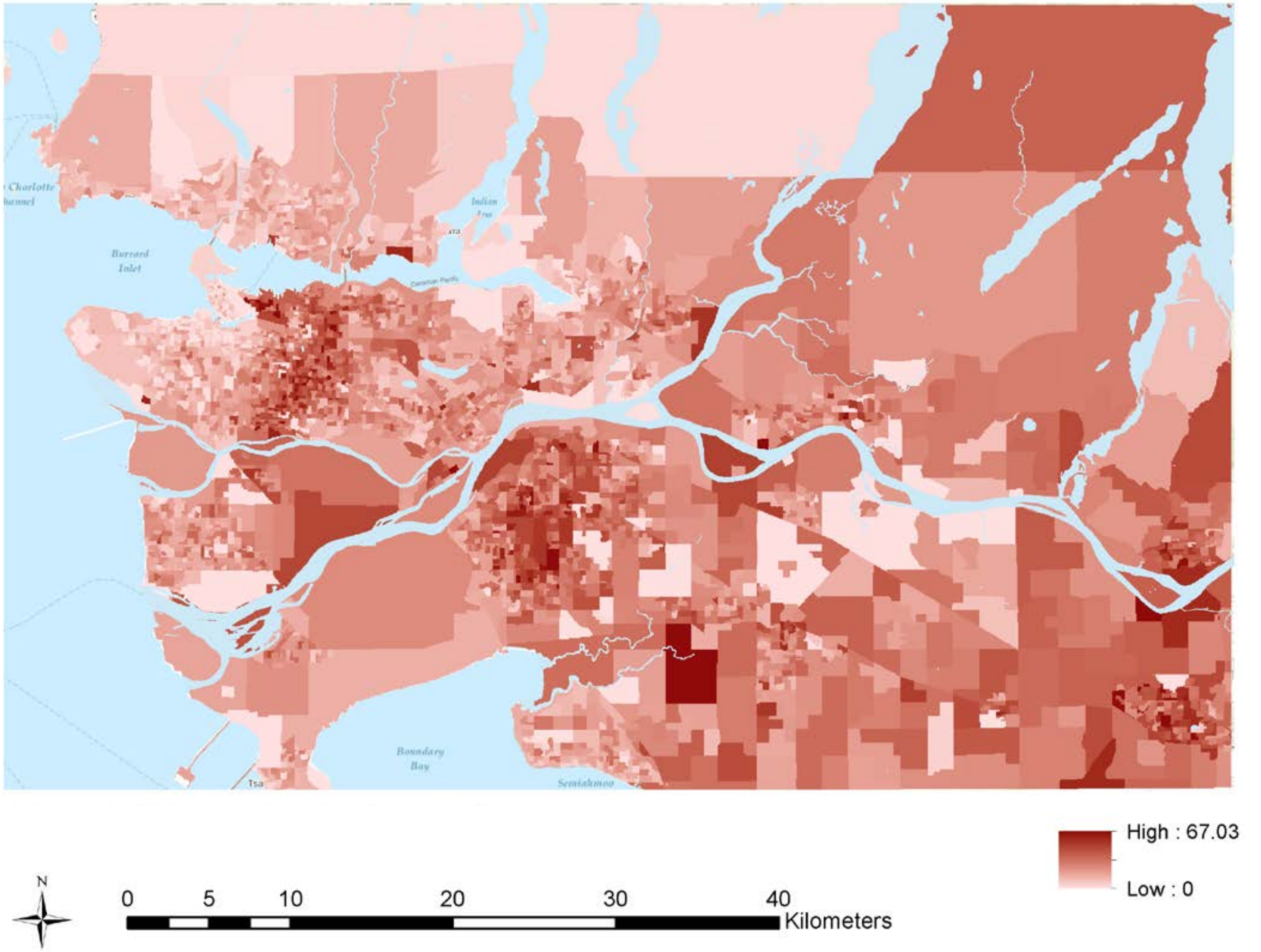

Figure S5. Map of the No High School (%) spatial variable

The variable was mapped using data from the 2006 national census, sourced from SimplyMap (Geographic Research Inc 2015).

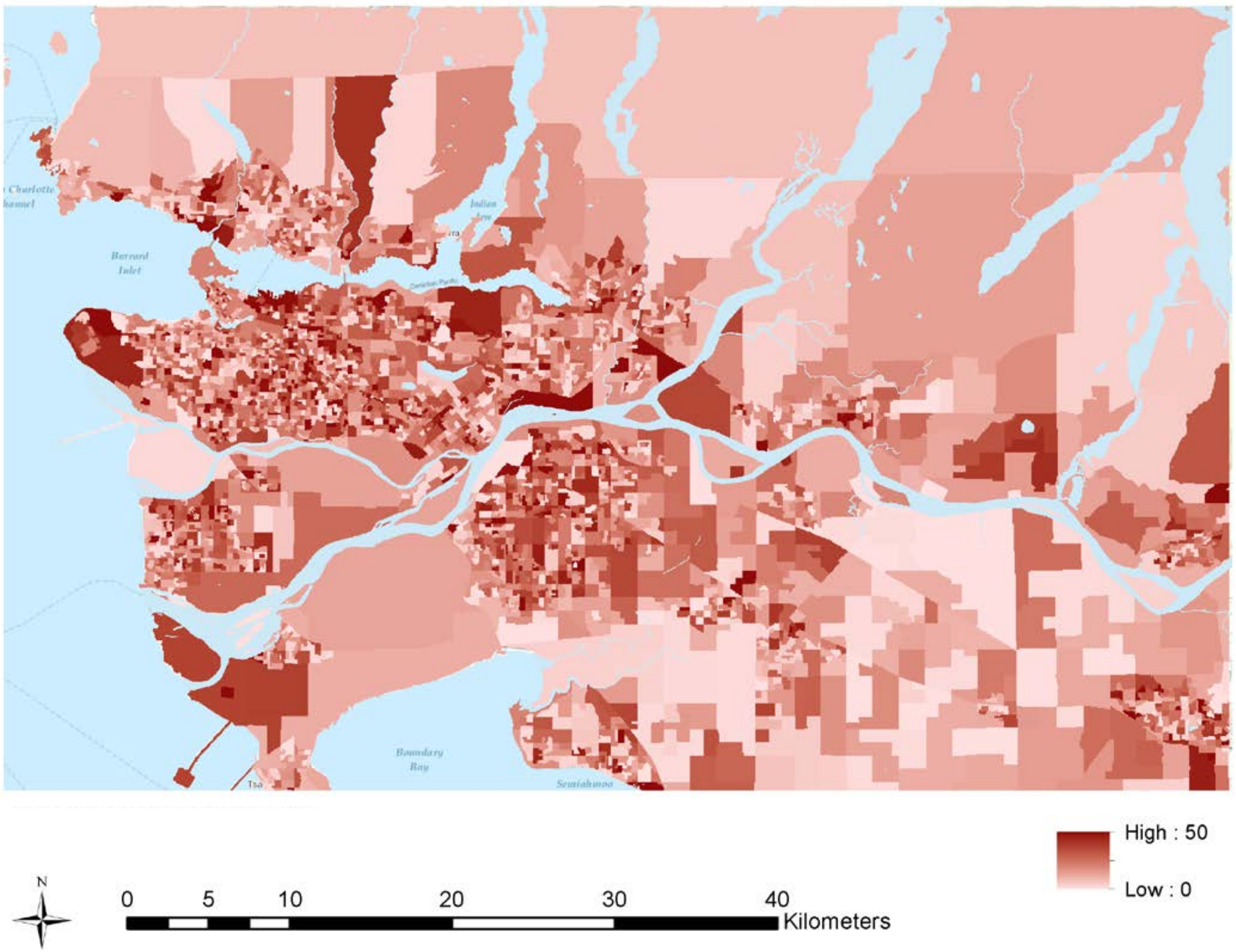

Figure S6. Map of the Unemployment Rate (%) spatial variable

The variable was mapped using data from the 2006 national census, sourced from SimplyMap (Geographic Research Inc 2015).

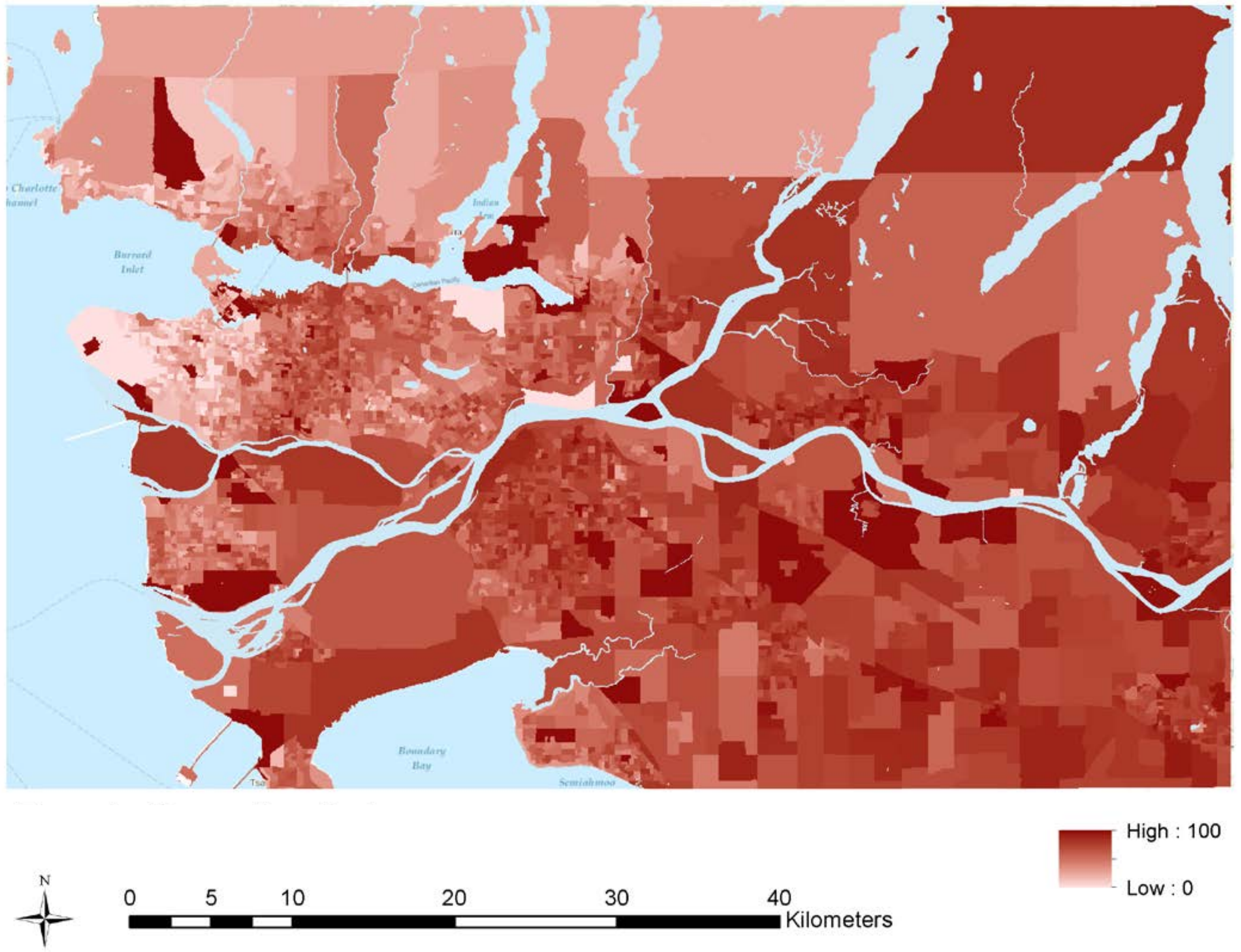

Figure S7. Map of the No University (%) spatial variable

The variable was mapped using data from the 2006 national census, sourced from SimplyMap (Geographic Research Inc 2015).

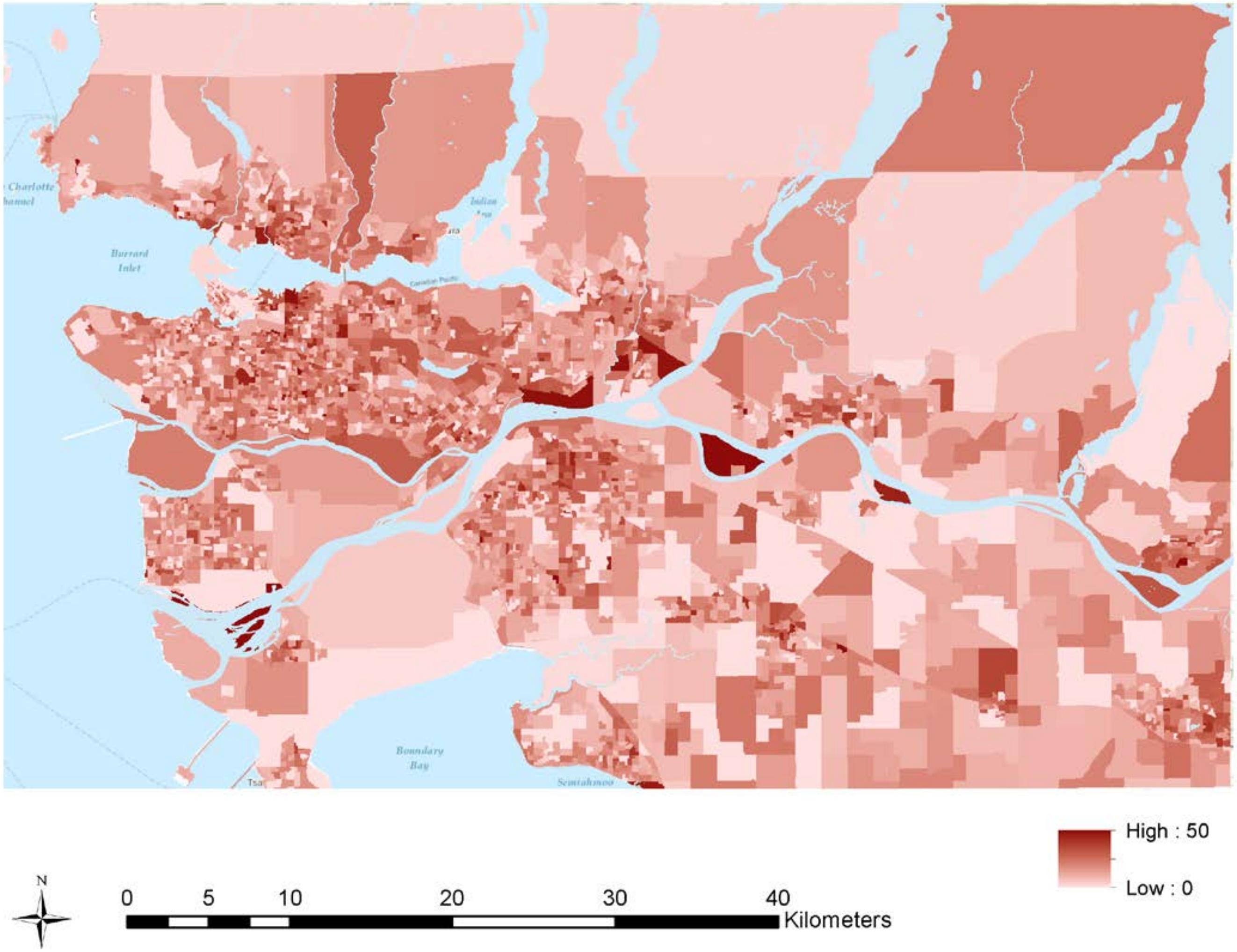

Figure S8. Map of the Single Parent Families (%) spatial variable

The variable was mapped using data from the 2006 national census, sourced from SimplyMap (Geographic Research Inc 2015).

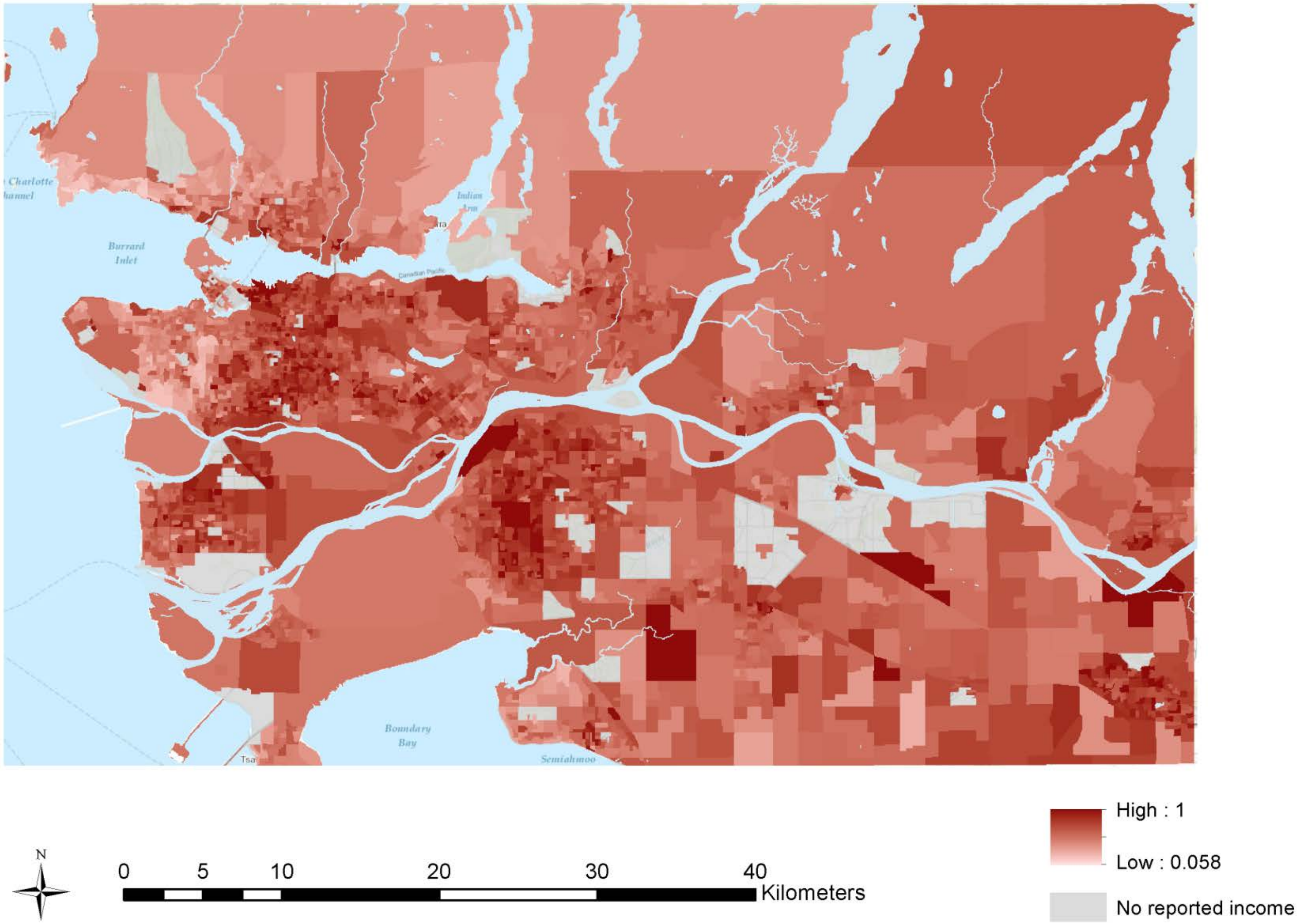

Figure S9. Map of the Average Income Ratio spatial variable

The variable was mapped using data from the 2006 national census, sourced from SimplyMap (Geographic Research Inc 2015).

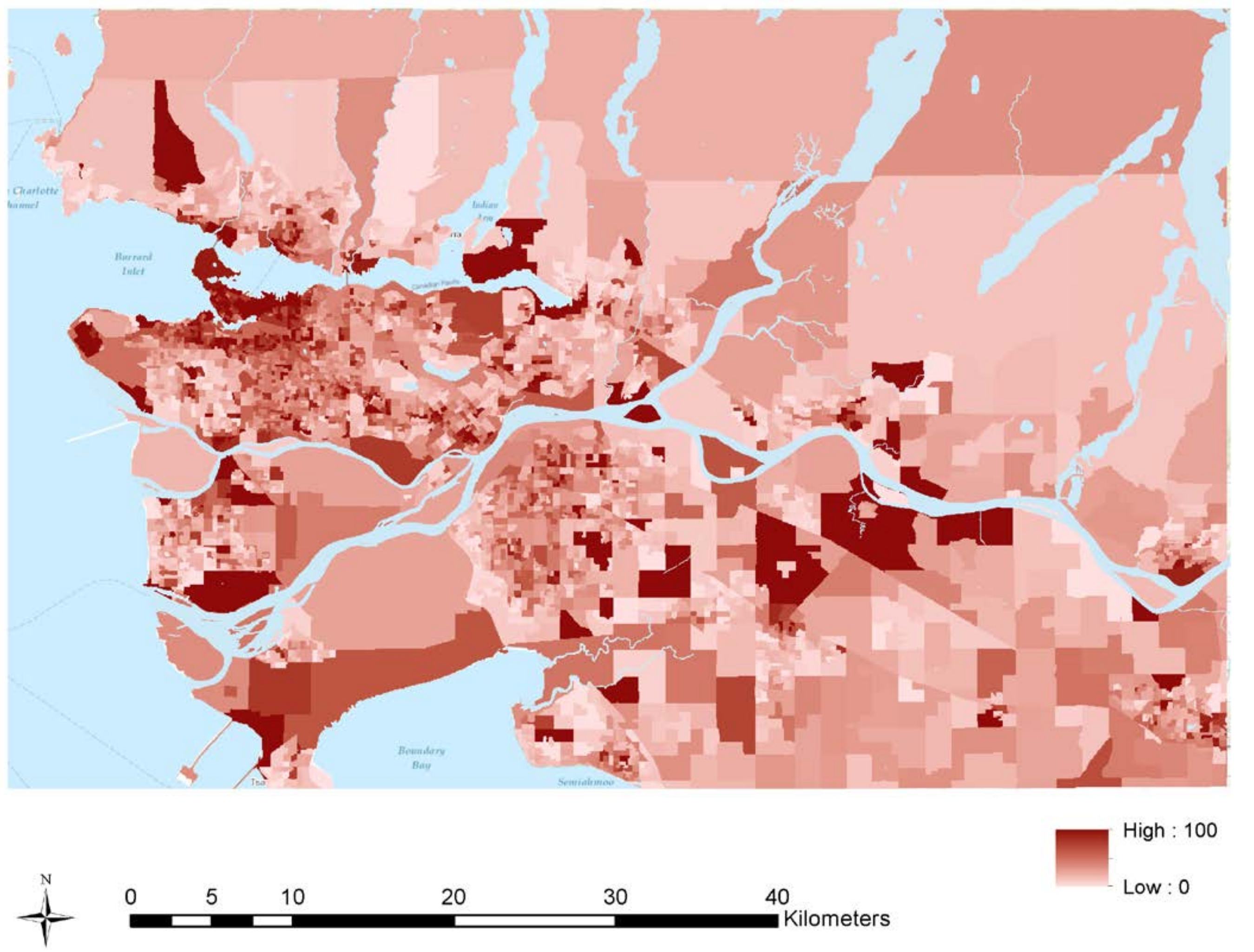

Figure S10. Map of the Homes Rented (%) spatial variable

The variable was mapped using data from the 2006 national census, sourced from SimplyMap (Geographic Research Inc 2015).

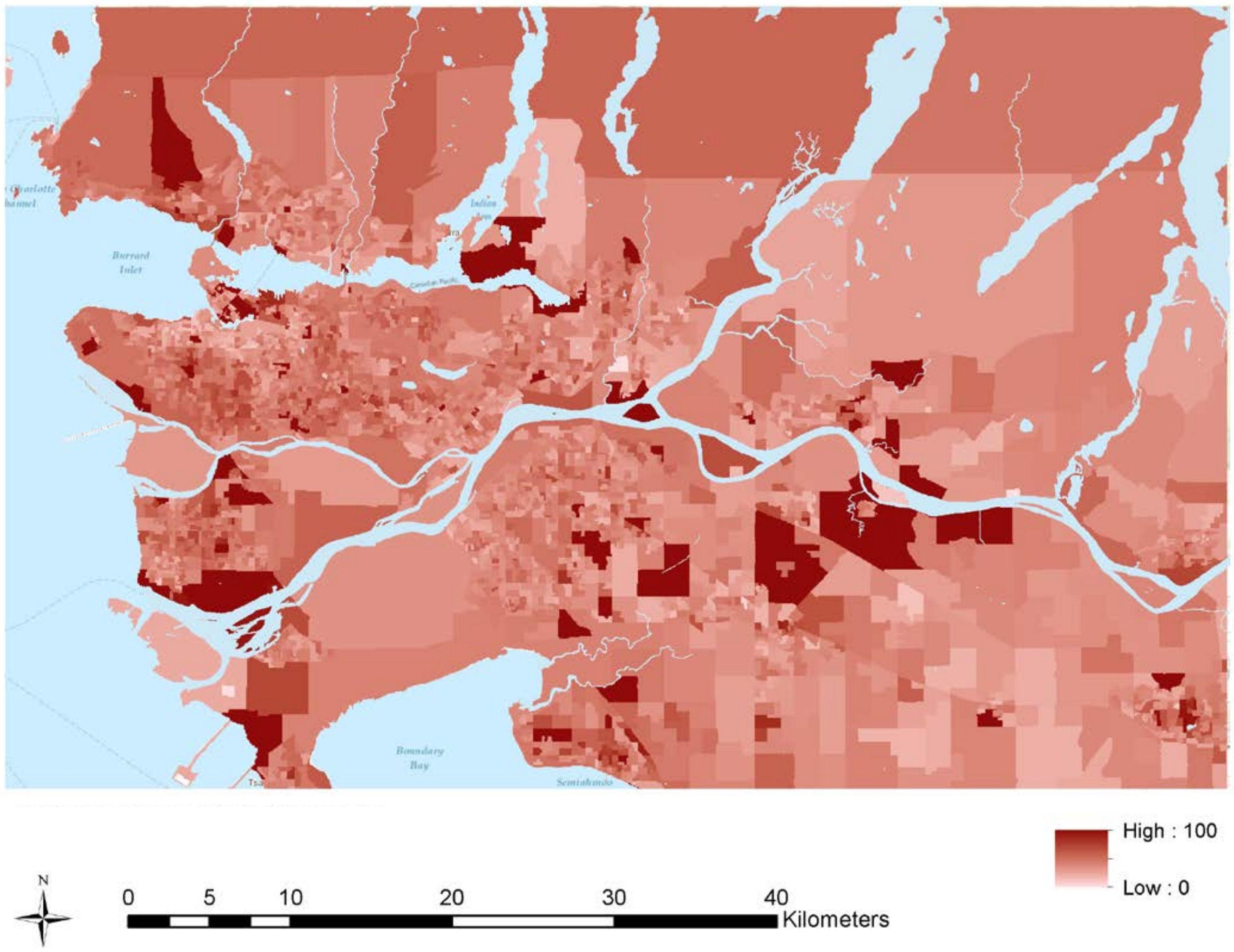

Figure S11. Map of the Labour Nonparticipation Rate (%) spatial variable

The variable was mapped using data from the 2006 national census, sourced from SimplyMap (Geographic Research Inc 2015).

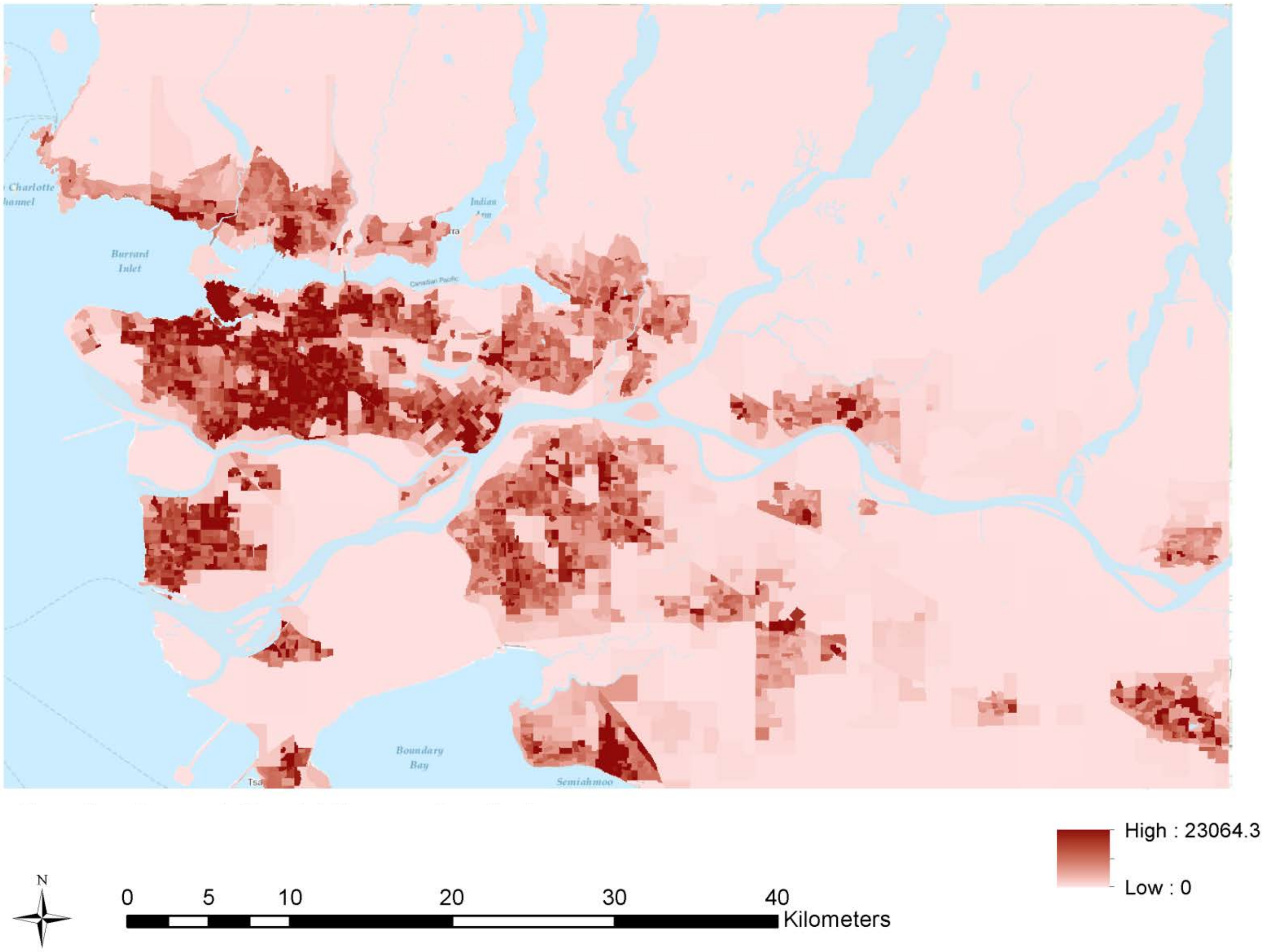

Figure S12. Map of the Density of Population ≥ 55 (km^{-2}) spatial variable

The variable was mapped using data from the 2006 national census, sourced from SimplyMap (Geographic Research Inc 2015).

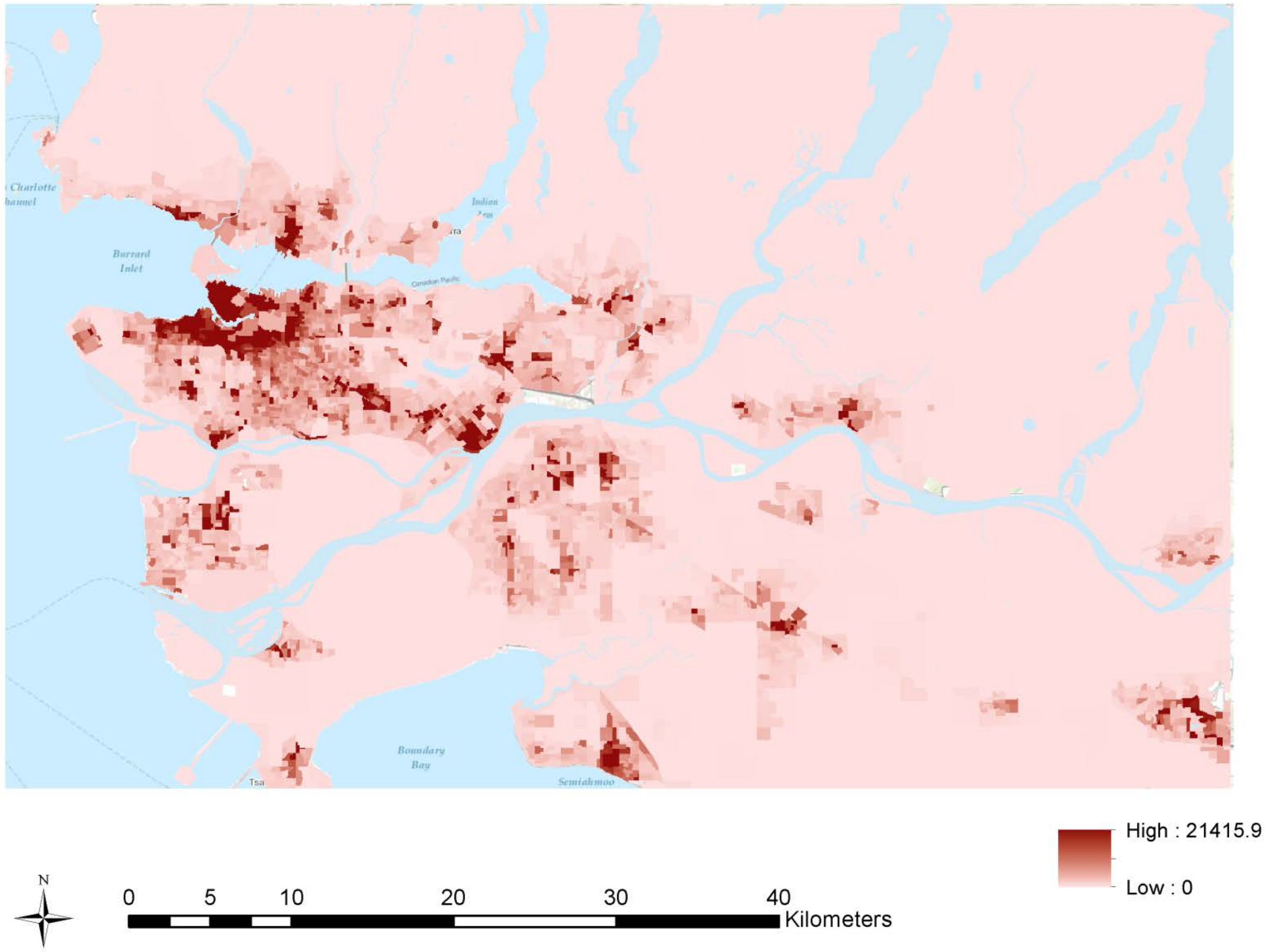

Figure S13. Map of the Density of Persons Living Alone (km^{-2}) spatial variable

The variable was mapped using data from the 2006 national census, sourced from SimplyMap (Geographic Research Inc 2015).

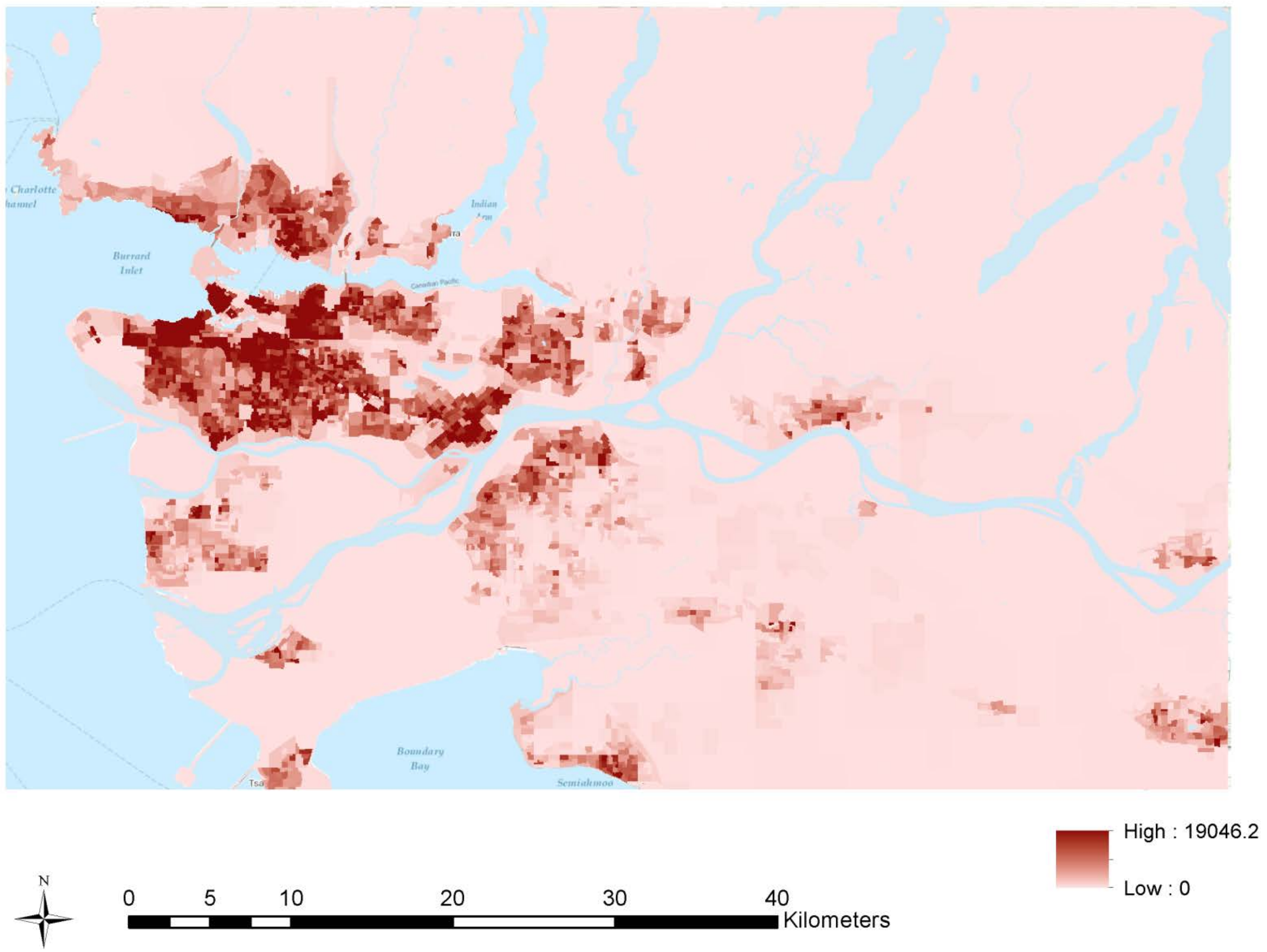

Figure S14. Map of the Density of Housing Built Prior to 1970 (km^{-2}) spatial variable

The variable was mapped using data from the 2006 national census, sourced from SimplyMap (Geographic Research Inc 2015).

References

Bell N, Schuurman N, Oliver L, Hayes MV. 2007. Towards the construction of place-specific measures of deprivation: a case study from the Vancouver metropolitan area. Can Geogr-Geogr Can 51(4): 444-461.

Bell N, Hayes MV. 2012. The Vancouver Area Neighbourhood Deprivation Index (VANDIX): a census-based tool for assessing small-area variations in health status. Canadian journal of public health = Revue canadienne de sante publique 103(8 Suppl 2): S28-32.

Geographic Research Inc. 2015. Census 2006 Current Estimates Data retrieved March 31st, 2015 from SimplyMap database.

Ho HC, Knudby A, Sirovyak P, Xu YM, Hodul M, Henderson SB. 2014. Mapping maximum urban air temperature on hot summer days. Remote Sens Environ 154: 38-45.

Ho HC, Knudby A, Xu Y, Hodul M, Aminipouri M. 2016. A comparison of urban heat islands mapped using skin temperature, air temperature, and apparent temperature (Humidex), for the greater Vancouver area. Sci Total Environ 544: 929-938.

Walker BB, Schuurman N, Hameed SM. 2014. A GIS-based spatiotemporal analysis of violent trauma hotspots in Vancouver, Canada: identification, contextualisation and intervention. Bmj Open 4(2).
